# Rain induces temporary shifts in epiphytic bacterial communities of cucumber and tomato fruit

**DOI:** 10.1038/s41598-020-58671-7

**Published:** 2020-02-04

**Authors:** Sarah M. Allard, Andrea R. Ottesen, Shirley A. Micallef

**Affiliations:** 1 Department of Plant Science and Landscape Architecture, University of Maryland, College Park, MD United States; 2 Division of Microbiology, Office of Regulatory Science, Center for Food Safety & Applied Nutrition, Food and Drug Administration, College Park, MD United States; 3 Center for Food Safety and Security Systems, University of Maryland, College Park, MD United States; 4 Present Address: School of Medicine, University of California, San Diego, CA United States

**Keywords:** Microbiology, Ecology

## Abstract

Understanding weather-related drivers of crop plant-microbiome relationships is important for food security and food safety in the face of a changing climate. Cucumber and tomato are commercially important commodities that are susceptible to plant disease and have been implicated in foodborne disease outbreaks. To investigate the influence of precipitation on plant-associated microbiomes, epiphytically associated bacterial communities of cucumber and tomato samples were profiled by 16 S rRNA gene sequencing (V1-V3) in the days surrounding two rain events over a 17-day period. Following rain, α (within-sample) diversity measured on cucumber and tomato fruit surfaces, but not tomato leaf surfaces, increased significantly and remained elevated for several days. Bacterial β (between-sample) diversity on cucumber and tomato fruit responded to precipitation. In the cucumber fruit surface (carpoplane), notable shifts in the families Xanthomonadaceae, Oxalobacteriaceae, Sphingobacteriaceae and Comamonadaceae were detected following precipitation. In the tomato carpoplane, shifts were detected in the families Enterobacteriaceae and Xanthomonadaceae following the first rain event, and in the Pseudomonadaceae and Oxalobacteriaceae following the second rain event. Few taxonomic shifts were detected in the tomato leaf surface (phylloplane). Exploring rain-induced shifts in plant microbiomes is highly relevant to crop protection, food safety and agroecology, and can aid in devising ways to enhance crop resilience to stresses and climate fluctuations.

## Introduction

Fruits and vegetables host distinct bacterial assemblages on various plant organs^1–3^. Plant microbiomes are dynamic and undergo successional changes with plant development^4^, possibly with new introductions occurring throughout the plant life cycle. Several bacterial reservoirs for the phyllosphere microbiome have been reported, including the air^5^, insect pollinators^6^, seed^7^, other nearby plants^5^, and meteorological conditions^8^. The impact of the latter on fresh produce crop microbiomes is of particular interest due to the highly variable nature of weather-related events, variation due to geography, and anticipated changes in precipitation patterns in the coming years due to climate change^9^. Increased precipitation and humidity often favor the development of plant disease^10,11^. Similarly, the prevalence of several foodborne pathogens including pathogenic *Escherichia coli*, *Campylobacter jejuni*, *Salmonella enterica* and *Bacillus cereus* has been correlated with elevated environmental temperature and humidity^12,13^. In trials assessing the fate of *E coli*, fecal coliforms and enterococci applied to the lettuce phyllosphere, bacterial decline rates were slower under moderate and regular rain patterns^14^. At the community level, rainfall events may coincide with drastic changes in the leaf surface microbiomes of canola plants, although changes due to plant development could be difficult to detangle^15^. Below ground, some soil microbial communities are influenced by drying and wetting frequencies, especially those not normally exposed to large fluctuations in soil moisture^16,17^.

Rain may shift the microbial profile of phyllosphere communities through direct seeding of microbes present in rainwater, splash from surrounding soil, increasing water availability for existing microbes, or by washing off loosely adhered epiphytes, creating opportunities for others to fill their former niche. Airborne biological particles, including bacteria and fungi, may act as ice or cloud nuclei, particles around which rain droplets form. Levels of bioaerosols are elevated during rain events^18^, and in fact, plants have been suggested as “cloud seeders”^19^. Airborne microbes, classified as bioaerosols, may be transferred to plant surfaces directly via rainfall or indirectly from standing water after rainfall. In fact, *Salmonella enterica* serovar Typhimurium is capable of aerosolizing from puddles and colonizing tomato plants following simulated rain events^20^. Rain splash dispersal can facilitate the transfer of human enteric bacteria from bulk soil to leaf and fruit surfaces^21^ even with the use of plastic mulch as a barrier^20^. Other microbes including plant pathogens are similarly capable of aerosolizing and retaining viability, sometimes incorporating aerosolization as part of their lifecycle^19,22^.

In the face of a changing and more variable climate, managing crop protection to ensure crop diversity, food security and food safety will necessitate a deep understanding of crop systems including their association with microorganisms and how they respond to external conditions and stresses. To garner a more comprehensive understanding of the impact of rain on the phytobiome of fresh produce crops that are vulnerable not only to plant disease but also colonization by human pathogens, we characterized the epiphytic bacterial communities dwelling on two commercially important fresh produce crops. A temporal assessment of the epiphytic bacterial communities of commercially cultivated cucumber fruit (carpoplane) following a rain event, and tomato carpoplane and leaves (phylloplane) surrounding two rain events was conducted.

## Materials and Methods

### Sample collection

**Figure 1:**
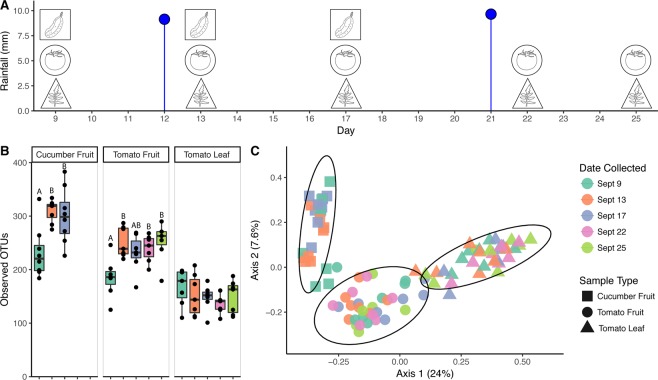
Sample type collection scheme and bacterial community profiles. A: Cucumber fruit (squares), tomato fruit (circles) and tomato leaf (triangles) samples were collected on the marked dates in September 2015 (n = 8 cucumber fruit, n = 7 tomato fruit, n = 7 tomato leaf per date), surrounding 2 rain events marked with blue lollipops. B. Boxplots representing Observed OTUs classified at 97% identity counted from rarefied dataset (8,200 seqs/sample) between sample types and across sampling dates. Lower and upper hinges show the 25^th^ and 75^th^ percentiles of the range, and whiskers extend to values furthest from the median that fall within 1.5× the interquartile range. Outlying points as well as inlying points for each sample are shown as dots, and the median is marked with a horizontal line in each box. Within each sample type, differential letter assignment indicates significant difference by date of collection. C. Principal Coordinates Analysis plot showing the clustering of samples as measured by Unweighted UniFrac distance, with 95% confidence ellipses surrounding each of the 3 sample types.

Samples were collected from an established Maryland farm under agricultural cultivation for 40 years. Tomato (*Solanum lycopersicum* cultivar ‘Christa’) and cucumber (*Cucumis sativus* cultivar ‘Sweet Success’) plants were grown for commercial purposes on black plastic mulch and drip irrigated throughout the season. Pest control and fertilizer management were conducted according to typical management practices for the farm. Samples were collected over a 17-day period in September 2015, on 5 dates surrounding 2 rain events on 9/12 and 9/21. Sampling occurred on 9/9 (pre-rain), 9/13 (1 day post-rain), 9/17 (5 days post-rain), 9/22 (1 day post-rain; tomato fruit and leaves only) and 9/25 (4 days post-rain; tomato fruit and leaves only) (Fig. 1A). At each timepoint, ripe tomato fruit (n = 7, 3 fruit per sample) and leaves (n = 7, 2 compound leaves per sample) and ripe cucumber fruit (n = 8, 1 fruit per sample, first 3 dates only) were aseptically collected in Ziploc bags and transported on ice in a cooler to the lab, where they were stored at 4 °C until processing within 24 h.

### DNA isolation and amplicon sequencing

Sterile deionized water was added to sample bags (100 ml for tomato fruit and leaves, 50 ml for cucumbers). Submerged samples were hand massaged through the bag for 30 s then sonicated in a Branson Ultrasonic Bath 8510 (Branson Ultrasonics, Danbury, CT) for 3 min at a frequency of 40 kHz to dislodge bacterial cells from the carpoplane and phylloplane. Samples were hand massaged again and sonicated for an additional 3 min before filtration. Carpoplane and phylloplane washes were filtered through sterile 0.22 µm nitro-cellulose filters (Nalgene, Rochester, NY), and filters were frozen at −80 °C until further processing. Total community DNA was extracted from filters using the MoBio PowerWater kit (MoBio Laboratories, Carlsbad, CA). The V1-V3 region of the 16 S rRNA gene was chosen for use in bacterial community profiling using 8F-533R primers^3^. Sequencing was carried out using 300-bp paired-end sequencing on the Illumina MiSeq (v3). Illumina’s protocol for 16 S Metagenomic Sequencing Library Preparation was followed for all samples (Illumina part # 15044223 rev. B) as previously described^23^.

### Sequencing data analysis

Quality filtering and sequence analysis were carried out using QIIME v. 1.8^24^, Mothur v. 1.34^25^, and Phyloseq v. 1.24.0^26^ in R v. 3.5.0. Prior to alignment, sequences went through several quality filtering steps to remove chimeras^27^, non-target sequences (chloroplast and mitochondria), and sequences less than 100 bp in length. Sequences were aligned to the Greengenes Core Set^28,29^ using PyNAST^30^, and taxonomy assignment utilized the RDP Classifier 2.2^31^. Reads that failed to match the reference database were clustered *de novo* using UCLUST v. 1.2.22^32^.

To ensure comparability between samples, the dataset was subsampled to the lowest common sequencing depth, 8,200 sequences per sample. Sample types (cucumber fruit, tomato fruit, tomato leaves) were analyzed separately to assess the influence of rainfall events on bacterial diversity for each of these niches. Beta (between sample) diversity was assessed using both unweighted distance matrices and matrices weighted by relative taxon abundance. Phylogenetic distance was incorporated into both distance matrices using UniFrac^33,34^. Bray-Curtis dissimilarity, which includes abundance but not relatedness in calculation of dissimilarity^35^, was also assessed. Adonis (999 permutations), a nonparametric MANOVA from R’s Vegan package, was implemented to assess significance of treatment influence on bacterial community structure. Using Principal Coordinates Analysis (PCoA) generated through R’s Vegan and Phyloseq packages, plots were created to visualize β-diversity. Alpha (within sample) diversity was assessed on the rarefied dataset in Phyloseq using both Observed OTUs and Shannon Index metrics. Observed OTUs represent the number of unique Operational Taxonomic Units at 97% sequence similarity, while the Shannon Index takes into account the proportional abundances of the observed OTUs^36^. To compare α-diversity between groups of samples, ANOVA was employed followed by Tukey’s HSD tests for pairwise comparisons, in R’s stats package v. 3.5.0. More than 95% of sequences were identified to the family-level and differential abundance analysis was continued at this taxonomic rank for the unrarefied dataset using DESEQ. 2^37^.

Sample information, sequence data and contingency matrices are available with Qiita study id 12262 (https://qiita.ucsd.edu). Sequence data have been deposited in the European Nucleotide Archive at the European Bioinformatic Institute under accession number ERP 118277.

### Weather data

Daily precipitation measurements were obtained from the National Oceanic and Atmospheric Administration website (www.noaa.gov), using climatological data collected from a weather station located 9 km from the sample site. Only limited weather data was available from the local station, so additional weather measurements, including temperature, barometric pressure, and wind speed, were acquired an Automated Weather Observing Station located 18 km away. The weather station is operated by the Federal Aviation Administration and administered by NOAA (National Centers for Environmental Information, Asheville, NC), and data was accessed through Weather Underground (www.wunderground.com).

### Generic E. coli

For quantification of generic *E. coli* on plant surfaces, bags containing cucumber fruit received 50 ml of sterile water, and tomato fruit and leaves received 100 ml of sterile water. Sample bags were hand-massaged for 30 s each, and samples diluted in serial 10-fold steps. One ml aliquots of water from each bag, along with dilutions −1, −2, −3, and −4 were dispensed onto 3 M E. coli/Coliform Petrifilms (3 M, Maplewood, MN, US) and incubated overnight. After 24 h incubation at 35 °C, blue colonies with associated gas bubbles were counted and recorded as presumptive *E. coli*. For each dilution, the plate containing 15–150 colonies was selected for concentration calculation. Statistical analysis was conducted in the R stats package v. 3.5.0 using ANOVA and Tukey’s HSD tests.

## Results

### Sequencing metrics

Approximately 4 million raw reads from 94 multiplexed samples (roughly 46,605 ± 15,136 reads per sample) with an average length of 463 bases, and an average Q score of 35 were further filtered for quality. Reads 1 and 2 were merged at an average efficiency of 83%. High quality unmerged read 1 was also included in downstream analysis. All samples had a Good’s Coverage value exceeding 0.95, indicating that samples were sequenced to a level nearing saturation^38^. After rarefaction, 770,810 sequences were retained for the final analysis.

### Weather

A dry period had been recorded prior to the commencement of sampling, with the most recent precipitation dating back to a 21 mm rain event on 8/20, 3 weeks prior to the first pre-rain sampling. The first rain event (Rain 1) on 9/12 recorded 9.14 mm of precipitation and the second event (Rain 2) on 9/21 reached 9.65 mm (Fig. 1). The highest daily temperature was recorded on 9/9 and a wind gust occurred on 9/13 around sampling time. Barometric pressure was low on 9/9 and 9/13 compared to the other sampling dates. Rain 1 was accompanied by thunder and lightning.

### Cucumber carpoplane

**Figure 2:**
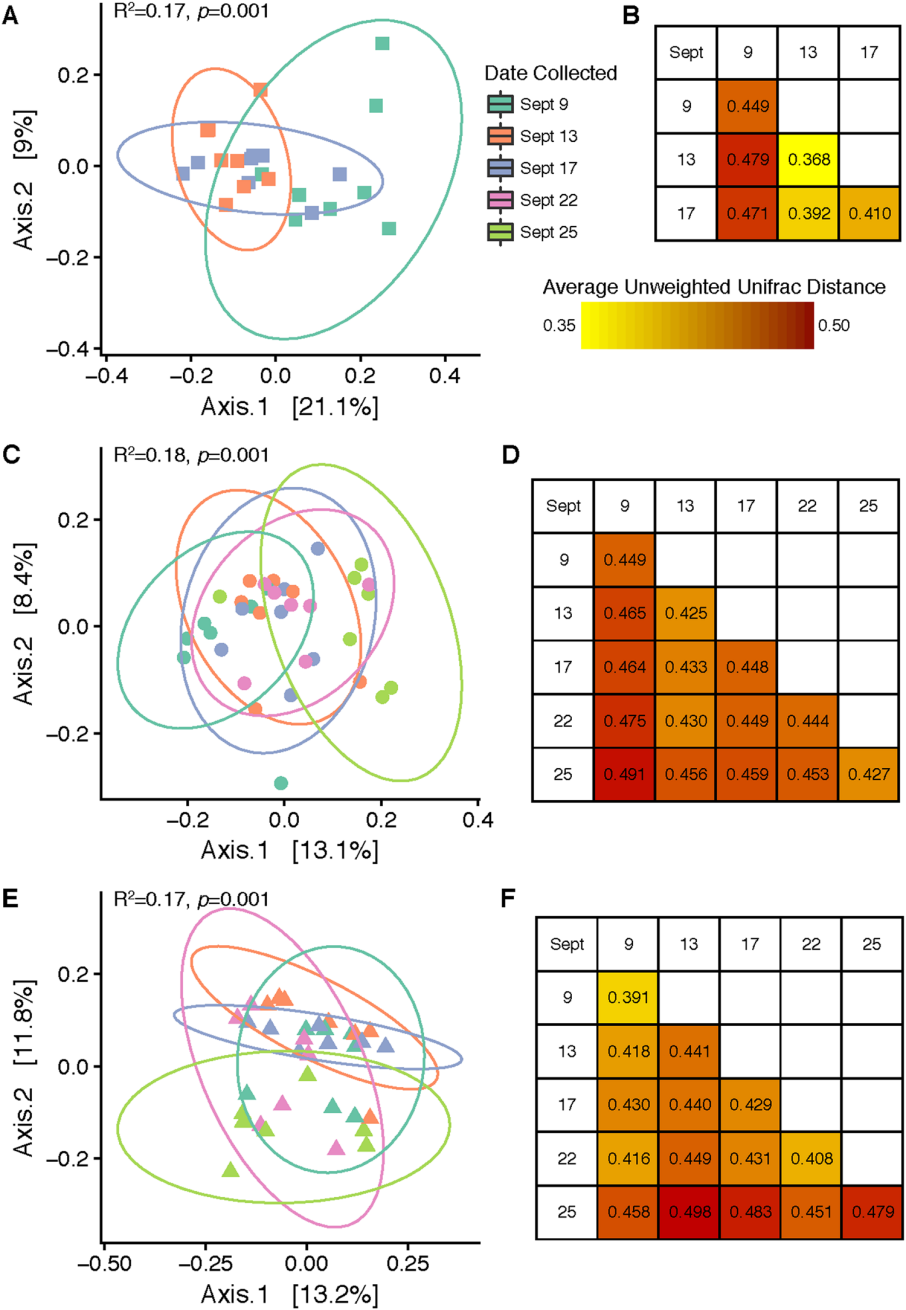
Influence of rain on cucumber and tomato-associated bacterial communities. Panels A, C and E show PCoA plots created from unweighted (including taxonomic identification but not relative abundance) UniFrac distance matrices comparing bacterial community β-diversity before, 1 day after, and 4–5 days after two rain events on the surfaces of Cucumber Fruit (A), Tomato Fruit (C) and Tomato Leaf (E). Panels B, D and F display the average unweighted UniFrac distances between sample groups collected on same or different days for each sample type (B = Cucumber Fruit, D = Tomato Fruit, F = Tomato Leaf).

Following the 9/12 rain event (Rain 1), a significant change in cucumber fruit surface bacterial community structure was observed for both unweighted UniFrac (*R*^2^ = 0.173, *p* = 0.001) and weighted UniFrac (*R*^2^ = 0.225, *p* = 0.005) analyses (Fig. 2A, Figure S1). Cucumber fruit samples collected 3 days prior to Rain 1 supported bacterial communities that clustered separately from those collected 1 day after Rain 1, and the largest average unweighted UniFrac distances were obtained between samples collected on 9/9–9/13 and 9/9–9/17 (Fig. 2B). Although some samples collected on 9/17 generated bacterial community profiles resembling the pre-rain profile, some resembled the immediate post-rain profile, a trend evident across 3 different distance metrics (Figure S1). Therefore, within 4 days a partial return to the pre-rain profile was detected.

**Figure 3:**
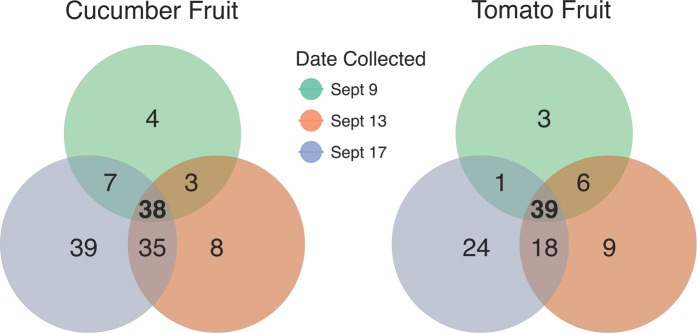
Bacterial OTUs associated with cucumber and tomato carpoplanes on 3 dates surrounding Rain 1. OTUs present in 100% of samples collected on 1, 2, or all 3 dates from 9/9 to 9/17 were tallied and represented in a Venn diagram. The list of ephemeral (present on only 1 or 2 dates) taxa summarized here can be found in Supplementary Table 1.

On the cucumber carpoplane, α-diversity increased significantly following Rain 1, as measured by both Observed OTUs (*p* = 0.004) and Shannon Index (*p* = 0.002), escalating from an average of 232 ( ± 48) to 310 ( ± 21) OTUs per sample, a 33.6% increase (Fig. 1B). Five days after Rain 1, α-diversity remained elevated compared to pre-rain levels (*p* = 0.01 and *p* = 0.06 for Observed OTUs and Shannon Index, respectively), with an average of 300 ( ± 52) OTUs per cucumber. Many of these OTUs were introduced across all replicates, indicating a common source. A core microbiome analysis was conducted to identify taxa shared by 100% of samples collected on each date and across multiple dates. Thirty-eight OTUs were ubiquitous among all dates. Seventy-four OTUs not observed in pre-rain cucumber carpoplane samples were identified in samples collected 1 day post-rain (Fig. 3, Supplementary Table 1). Of these, 35 OTUs were retained across all replicates 4 days later. By contrast, only 7 OTUs observed in pre-rain samples were not detected 1 day post-rain, with 3 of these being observed again 5 days post-rain.

**Figure 4:**
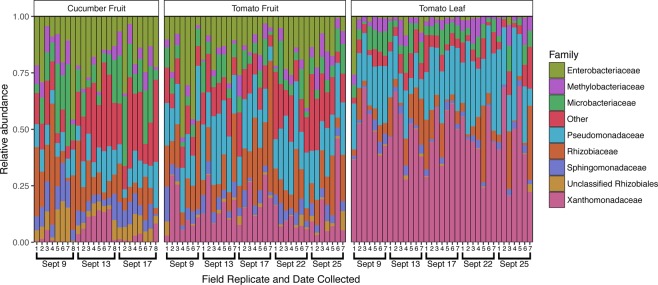
Family-level taxonomic profiles of all sample types across the study period. The top 8 most relatively abundant taxa are represented individually, with all other taxa grouped into the “Other” category, with all samples rarefied to 8,200 sequences. Sampling dates and replicate numbers for all sample types are marked: cucumber fruit (n = 8), tomato fruit (n = 7), and tomato leaf (n = 7).

In addition to the introduction of new taxa, changes in the relative abundance of established taxa on the cucumber surface were observed following Rain 1 (Fig. 4). Of 809 total OTUs in the cucumber dataset, 112 were differentially abundant between samples collected on Sept 9 and 13 (pre- and post-rain). At the family level, 16 bacterial families differed between the pre- and post-rain timepoints. Notably, the family Xanthomonadaceae increased from an average of 1.2% to 9.6% relative abundance following rain (adjusted *p* < 0.001), dropping to 2.4% 5 days after rain. The Oxalobacteriaceae exhibited a similar increase, from 0.6% to 7.0% (adjusted *p* < 0.001), but in this case average relative abundance remained high after 4 days, at 7.1%. Similarly, the Sphingobacteriaceae and Comamonadaceae, initially detected at less than 0.2% average relative abundance, increased at least an order of magnitude in relative abundance following rainfall, remaining elevated 4 days later (adjusted *p* = 0.005 and *p* < 0.001, respectively). Relative abundance for several of the most dominant bacterial families on the cucumber surface declined or increased following rainfall. The Sphingomonadaceae decreased from an average of 9.6% to 5.1% relative abundance following rainfall (adjusted *p* = 0.001) but increased 4 days later to 7.6%. Similarly, the average relative abundance of the family Microbacteriaceae diminished following rainfall (from 15.6% to 8.6%, adjusted *p* = 0.003), increasing to an even higher average relative abundance later (17.9%). The Enterobacteriaceae demonstrated an opposite shift that was not significant (adjusted *p* = 0.323), increasing 1 day after rain from 18.4% to 21.9%, later returning to 17.2% average relative abundance (Fig. 4). Although these changes in relative abundance are indicative of community shifts, they do not necessarily translate to increases or decreases in the absolute abundance of certain taxa.

### Tomato carpoplane

Two rain events were followed in analysis of tomato bacterial communities. This may be the reason why β-diversity of the tomato carpoplane did not strictly parallel the same trend observed on cucumber fruit. There was an overall effect of sampling date on bacterial communities when analyzed using unweighted UniFrac distance (*R*^2^ = 0.177, *p* = 0.001; Fig. 2C) and Bray-Curtis dissimilarity (*R*^2^ = 0.186, *p* = 0.005) but not weighted UniFrac distance (*R*^2^ = 0.136, *p* = 0.225) (Figure S1). Throughout the sampling period, unweighted UniFrac distance increased steadily in comparison to the first pre-rain sampling date, with the greatest distance measured between samples collected on 9/9 (pre-rain) and 9/25 (post-Rain 2) (Fig. 2D).

On the other hand, following Rain 1, α-diversity on the tomato carpoplane resembled the dynamics seen on cucumbers. Observed OTU count increased from 185 ( ± 37) to 251 ( ± 28) OTUs per sample (*p* = 0.005) from 9/9 to 9/13 (Fig. 1B). Five days after Rain 1, α-diversity by both measures was indistinguishable from pre-rain and 1 day post-rain levels (*p* > 0.05), at 231 ( ± 35) OTUs per sample. Following Rain 2, observed OTU count remained elevated compared to pre-Rain 1 (*p* = 0.03 and *p* = 0.004 for 9/22 and 9/25, respectively) but did not significantly increase beyond 9/13 levels. Analysis of the same dates by Shannon Index suggests that across the sampling period, α-diversity was different only between 9/9 and 9/13 (*p* = 0.058). Following Rain 1, similar trends in the core microbiome as those on the cucumber carpoplane were observed on tomato fruit. For the 3 dates surrounding Rain 1, tomato fruit collected on each day supported OTUs common to all samples collected on that date but not observed on any other date, with 9/17 hosting the most unique core OTUs (Fig. 3, Supplementary Table 1). Moreover, 42 additional OTUs were present across all post-Rain 1 samples, of which 18 persisted in all samples 4 days later. Following Rain 2, only 17 new OTUs were detected on all tomato fruit samples, 7 of which remained present on all samples on the final sampling day.

Following Rain 1, the relative abundance of 44 OTUs changed significantly on the tomato fruit surface, including 10 members of the Enterobacteriaceae and 3 members of the Xanthomonadaceae, all of which increased in average relative abundance. There were no significant differences at the family level between 9/9 and 9/13, however there were significant differences in 6 low-abundance ( < 5%) bacterial families between 9/9 and 9/25. For example, relative abundance of the family Rhodobacteriaceae increased steadily over the study period (*p* = 0.006), from ~0.35% to 3.5%. The most abundant taxa tended to fluctuate throughout the study period irrespective of proximity to rainfall. On average, the Pseudomonadaceae increased in relative abundance from 9/9 (18.2%) to 9/13 (23.7%), decreased on 9/17 (16.9%), only to increase again after Rain 2 (23.4%) and decrease again by 9/25 (19.6%). The increase from 9/9 to 9/13 was not statistically supported, however the increase from 9/17 to 9/22 was statistically significant (adjusted *p* = 0.043). Relative abundance of the Oxalobacteriaceae also increased significantly following Rain 2, from 0.5% to 2.9% (adjusted *p* < 0.001), following a small borderline insignificant increase after Rain 1, from 9/9 (0.4%) to 9/13 (1.2%) (adjusted *p* = 0.081). This temporary post-rainfall increase in relative abundance of the Oxalobacteriaceae mirrored results seen on cucumber.

### Tomato phylloplane

On tomato leaf surfaces, increases in α-diversity around rainfall were not discernible, with an average of 150 (±30) OTUs per sample. Though not significant, the pre-rain timepoint had the highest median number of OTUs per sample (Fig. 1B). A core microbiome of 27 OTUs was observed in all leaf samples. Twenty-one OTUs common to all pre-rain samples were not observed 1 day post-rain, and only 6 new OTU introductions common to all 1 day post-rain samples were detected. No OTUs were found to be common across all samples between 1 day and 4 days post-rain timepoints other than the core 27 common to all 3 dates, while 7 OTUs shared among all 4 days post-rain and pre-rain samples were recovered. On the other hand, shifts in β-diversity were detected across the full sampling period; timepoint significantly influenced bacterial community structure at a rarefaction level of 8,200 sequences per sample when analyzed with unweighted UniFrac distance (*R*^2^ = 0.175, *p* = 0.001) and Bray-Curtis dissimilarity (*R*^2^ = 0.195, *p* = 0.036). However, weighted UniFrac analysis did not reveal a significant effect (*R*^2^ = 0.106, *p* = 0.521) (Fig. 2E, Figure S1). No taxa shifted in relative abundance between the pre-rain and 1 day post-rain timepoints, but changes in the average relative abundance of some low-abundance families (<0.2%) were detected between the pre-rain timepoint and 4 days post Rain 2. The Unweighted UniFrac distances between averaged sample groups from each timepoint suggested a weak but consistent shift in community structure over the course of the sampling period, but also considerable variation within samples from the last timepoint (9/25) (Fig. 2F).

### Cucumber versus tomato fruit

Cucumber fruit surfaces yielded a higher number of bacterial OTUs, 281 (±54), compared to tomato fruit surfaces, 232 (±40) (*p* < 0.001, Fig. 1B). Similarly, bacterial community structure differed significantly between the 2 fruit types (*R*^2^ = 0.246, *p* = 0.001) (Fig. 1C). Several of the most dominant taxa differed in average relative abundance between cucumber and tomato fruit surfaces, including Pseudomonadaceae (10.1% on cucumber, 19.6% on tomato, adjusted *p* < 0.001), Xanthomonadaceae (4.4% on cucumber, 15.5% on tomato, adjusted *p* < 0.001), Methylobacteriaceae (5.4% on cucumber, 3.5% on tomato, adjusted *p* = 0.038), and Microbacteriaceae (14.0% on cucumber, 6.7% on tomato, adjusted *p* < 0.001). Enterococcaceae were higher in average relative abundance on cucumber compared to tomato fruit (0.01% on cucumber, 0.002% on tomato, adjusted *p* = 0.003). Taxa of the Rhizobiaceae family were prevalent on both tomato (18.5%) and cucumber (13.7%) fruit surfaces and were not significantly different (adjusted *p* = 0.200). Similarly, members of the Enterobacteriaceae were dominant on both tomato (18.2%) and cucumber (19.2%) fruit surfaces (adjusted *p* = 0.932). (Fig. 4).

### Presumptive *E. coli*

**Figure 5:**
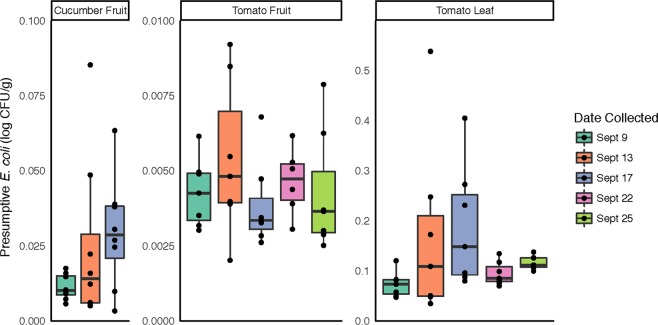
Presumptive *E. coli* recovery from cucumber and tomato surfaces. For each sampling date, serial dilutions from surface washes of the 3 sample types were dispensed onto selective media for quantification of colonies with morphology typical of generic *E. coli*.

In order to more directly address the food safety implications of potential bacterial community changes in response to rainfall, samples were screened for presumptive generic *E. coli*, frequently used as an indicator of fecal contamination. In total, tomato leaf samples had significantly higher levels of presumptive *E. coli* compared to tomato and cucumber fruit samples (*p* < 0.001; Fig. 5). No significant differences were observed between sampling dates for any sample type, although presumptive *E. coli* counts and variability in the data increased in all samples following Rain 1 and the highest individual values within each sample type were observed on 9/13, the day following the first rain event (Fig. 5).

## Discussion and Conclusions

Although the impact of precipitation on fruit and vegetable crop microbiomes has not been directly investigated, it has long been understood that increased plant disease and food safety risks can succeed rain events by enhanced dissemination of pathogens, splash or flooding and by provision of more favourable growth conditions^10,20^. Shifts in precipitation and periods of drought could also affect biocontrol microorganisms, altering the suppressive potential of microbiomes^39^. Reaching a more nuanced understanding of the precise effect of precipitation on crop microbiomes and the microbial dynamics that ensue is valuable to the application of systems thinking and approaches to crop protection, especially when confronted with climate change and increasing severity and duration of rainfall and drought periods. The consistent seasonal fluctuations recently described in the airborne microbiome captured in rain and snow^40^ imply that bacterial introductions via rain could be predictable during sequential rain events in a given agricultural region, hence allowing for crop management decisions to be made with regards to anticipated microbial dynamics in response to drought or precipitation.

In this study, assessment of microbiomes associated with cucumber and tomato surfaces using high quality amplicons of the 16 S rRNA gene provided a novel contribution to our understanding of the impact of rainfall on epiphytic bacterial communities at the time of crop harvest. This work demonstrated an increase in bacterial species diversity on cucumber and tomato fruit surfaces following rain events, at times accompanied by shifts in bacterial community structure. Several new bacterial taxa were introduced to the cucumber and tomato carpoplanes following rainfall and persisted at low abundance in the days following precipitation. This points to a window of time following precipitation during which introduced taxa may become newly established in the phyllosphere, potentially constituting a period of high risk for plant disease and food safety. On cucumber fruit surfaces, the relative abundance of several of the most dominant taxa changed following rainfall, often fully or partially returning to pre-rain proportions within 4 days. Furthermore, overall bacterial community structure on cucumber fruit shifted significantly in response to rain as measured both when incorporating phylogenetic relatedness of bacteria present (unweighted UniFrac) and including the relative abundance and phylogenetic relatedness of bacterial OTUs (weighted UniFrac). Tomato fruit-associated bacterial communities shifted throughout the study period when assessed by OTU richness and the unweighted UniFrac metric. By contrast, this observed sustained increase in diversity on the tomato fruit surface was not clearly accompanied by shifts in measures that take relative abundance into account, being only detected using Bray-Curtis dissimilarity but not weighted UniFrac. An additive effect considering multiple rain events could explain these differences, however other drivers cannot be ruled out. Phyllosphere microbiome responses to rain have not been investigated in crop systems but reports from rhizobacterial communities suggest that belowground microbiota may be more resilient^41^. Studies on the effect of precipitation on the rhizosphere microbiome appear to reveal that rain has a limited impact on bacterial community composition but can drive changes in relative abundance of taxa^42,43^. Our study indicated that phyllosphere bacterial assemblages of cucumber and tomato are responsive to precipitation events. This is turn suggests that phyllosphere microbiomes could be amenable to modulation with the aim of achieving desired outcomes such as disease resistance, enhanced food safety and stress tolerance.

Unlike fruit surface communities, tomato leaf surface community α-diversity remained largely consistent across all sampling dates; a decrease in the number of OTUs observed 1 day post-rain was not statistically supported. A shift in community structure was observed in the phylloplane over the course of the sampling period, however, in concordance with results from tomato fruit, the incorporation of abundance data into the UniFrac metric diluted the effect. For both tomato fruit and leaves, this indicates that any changes in community structure could likely be attributed to shifts in low-abundance taxa. In tomato fruit, these changes in low-abundance taxa could be observed both through shifts in relative abundance and introduced taxa. The diminished effect on leaves, on which little post-rain seeding of novel taxa was detected, could be the result of the strong influence of the plant host on species recruitment. However, abiotic conditions on the leaf could also be a factor, with higher relative humidity surrounding leaves as a result of transpiration and trapped moisture within the layer of abundant trichomes. By contrast, mature tomato fruit lack stomata and trichomes, such that moisture from rain could have significantly changed growth conditions by increasing the amount of free water available to microorganisms.

Compared to results seen on tomato, microbiota associated with the cucumber carpoplane were more responsive to weather-related changes, although only one rain event was evaluated in the case of cucumber. Fruit and vegetable crops harbor distinct bacterial communities^1^ that could be based on inherent differences in plant surface topography and nutritional profile. Differences in cropping practices could also partly explain differences between the two fruit crops. Cucumbers were grown on plasticulture on the ground, while tomatoes were staked upright. Cucumber fruit lying directly on plastic mulch were left both more exposed to direct rainwater contact and closer to the soil, increasing the potential for splash. Newly introduced taxa may have originated from rain or transferred via splash or wind from soil or nearby plant parts. While the sampling dates were selected to surround rain events, other weather dynamics during the sampling period could not be controlled for and likely influenced plant-associated microbiomes as well. Differences in barometric pressure and wind speed or reduced UV stress due to cloud cover could have influenced crop phytobiome dynamics or interacted with the factor of rain. Furthermore, rainfall may have been correlated with larger scale ecosystem changes. For example, insect visitation, which can affect plant microbiomes^23^ may have been limited during the rain event but elevated in the days following precipitation. Pesticides were applied to tomato plots during the sampling period, on the evenings of 9/9 and 9/16. It is possible that these applications could have influenced microbiome structure and diversity, however phyllosphere bacterial communities tend to be fairly robust in the face of pesticide application^44,45^. Pesticides were not applied to the cucumber plot and similar but more discernable responses to rainfall were detected on cucumber fruit.

Prior to Rain 1, the region experienced a long drought with negligible rainfall since the previous major rain event (21 mm) 3 weeks before the study began. The increase in diversity observed following Rain, 1 but not Rain 2, could be explained by drought-induced suppression of bacterial diversity at the start of sampling, not replicated prior to Rain 2, which occurred only 9 days later. Due to the close proximity of the rain events, it is possible that bioaerosols were less prevalent during the second rain. Plants release microbes into the atmosphere preferentially on sunny, dry days^46^, and there were few of those between the two rain events. Unfortunately, cucumber data for Rain 2 were not collected due to seasonality and a lack of availability of high-quality fruit samples, such that we cannot address whether the difference between Rain 1 and Rain 2 is mirrored on cucumber. The shifts in bacterial OTU richness and in some cases community structure that we did observe following rain events could have been the result of direct inoculation by rainwater-associated microbiota or by other factors associated with rainfall. Rain could physically remove microbes from the plant surface, opening up a niche for others to fill. Alternatively, increased moisture and relative humidity in the air before, during and after rain events could favor rapid growth of certain taxa at the expense of others. In previous work we noted that *E. coli* levels on lettuce spiked after moderate precipitation (20 mm) but plummeted after heavy rainfall^14^, suggesting that the effect of rain is a balance between new species introductions, stimulating growth conditions as a result of enhanced moisture, and a depleting effect, depending on rainfall depths. In this study, we saw an increase in variation of *E. coli* on fruit and leaves, but no significant hike in population levels, suggesting that this taxon is not an adequate indicator of bacterial community shifts in the phyllosphere.

While it is important to understand the local influence of isolated rainfall events on microbial dynamics in agriculture, in the future it will also be important to consider the influence of weather patterns on a larger scale. In addition to the direct impact of rainfall on phytobiomes, prolonged wet or dry periods could influence plant health and immune responses, and storms could lead to wounding, creating opportunities for pathogens to infiltrate plant tissues. Climatic change could lead to expanded ranges for plant pathogens due to favorable wet and warm conditions in higher latitudes and altered dispersal patterns influenced by intensified rain and wind, and shifting vector habitats^47^. Many factors associated with climate change, including elevated temperatures, increased periods of drought, intensified storms and elevated CO_2_ will likely influence the health of our crops directly^48^, but also indirectly by shifting the balance of microbes that may inhabit them, including plant pathogens, human pathogens and beneficial or commensal organisms. For some plant-pathogen pairs, weather-based forecasting models are already in use, helping growers time pesticide applications efficiently for the highest effectiveness and lowest environmental impact^49–51^. Similar decision support systems could be implemented for use in food safety, providing recommendations to growers on safest harvest times following single and repeated rain events. The first step in achieving this goal is amassing an understanding of the community-level dynamics on harvestable fresh produce preceding and following rain events.

## Supplementary information

Supplementary information.
