# Supplementary material for: Rain induces temporary shifts in epiphytic bacterial communities of cucumber and tomato fruit
Source: Sci Rep. 2020 Feb 4;10:1765. doi: 10.1038/s41598-020-58671-7 (PMC7000718; doi:10.1038/s41598-020-58671-7)
Supplement: Supplementary file 1 — Supplementary information. [file 41598_2020_58671_MOESM1_ESM.docx]

**Rain induces temporary shifts in epiphytic bacterial communities of cucumber and tomato fruit**

Sarah M. Allard^a,b#^ , Andrea R. Ottesen^b^, Shirley A. Micallef^a,c*^

Department of Plant Science and Landscape Architecture, University of Maryland, College Park, MD^a^; Division of Microbiology, Office of Regulatory Science, Center for Food Safety & Applied Nutrition, Food and Drug Administration, College Park, MD^b^; Center for Food Safety and Security Systems, University of Maryland, College Park, MD^c^

*Address correspondence to Shirley A. Micallef

Email: [smicall@umd.edu](mailto:smicall@umd.edu); Tel: +1 301-405-4369; FAX: +1 301-314-9308

^#^Present address: Sarah M. Allard, School of Medicine, University of California, San Diego, CA


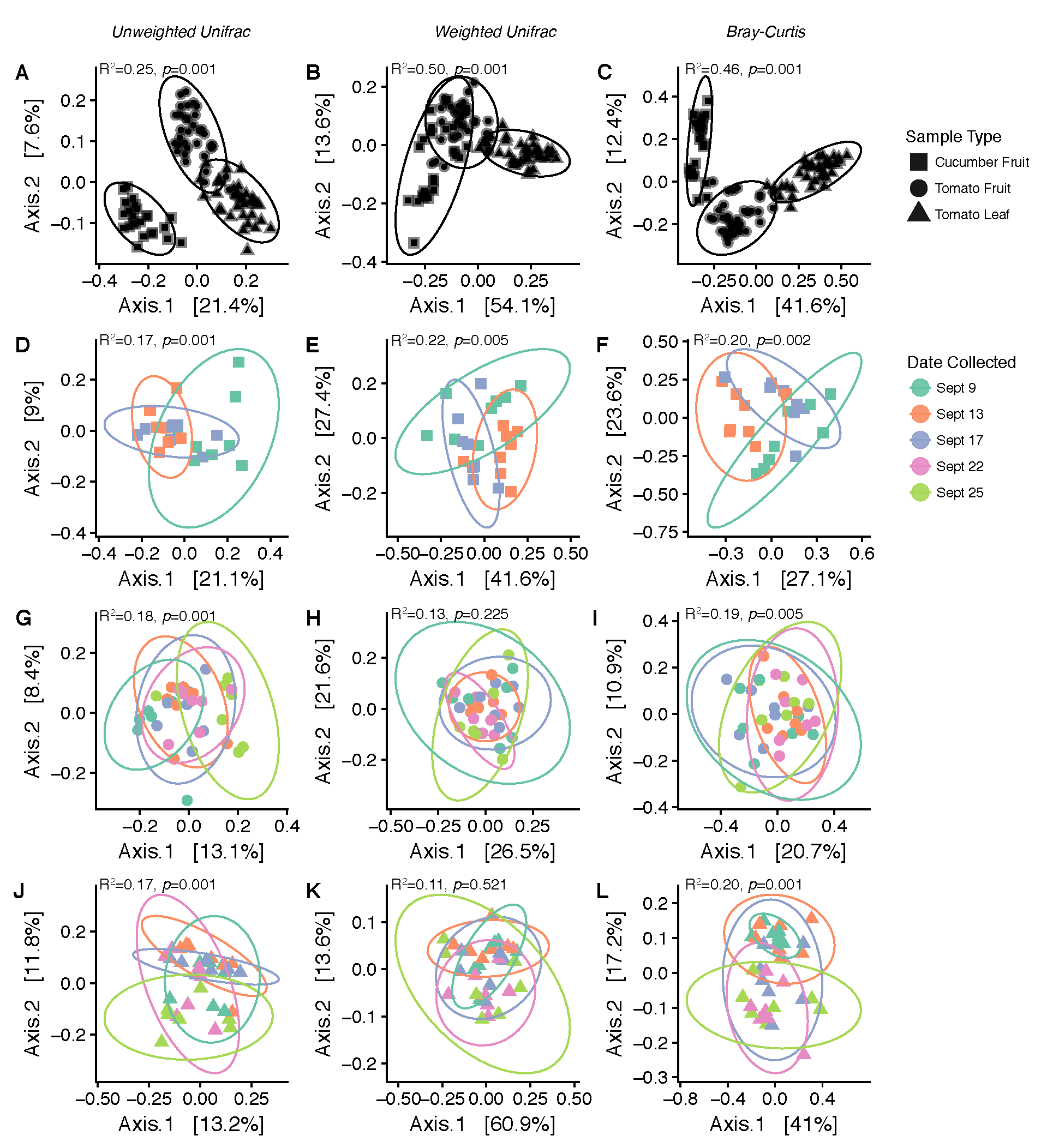


**Figure S1.** Βacterial β-diversity for all samples (A, B, C), cucumber fruit (D, E, F,), tomato fruit (G, H, I) and tomato leaves (J, K, L), as measured by unweigted UniFrac (A, D, G, J), weighted UniFrac (B, E, H, K) and Bray-Curtis dissimilarity (C, F, I, L).

**Supplementary Table 1.** Taxonomy of Core OTUs identified on 100% of cucumber or tomato fruit surfaces on each sampling day surrounding Rain 1, including those ephemeral taxa identified on only 1-2 dates and core taxa detected across all 3 dates (9/9, 9/13, 9/17).

| **Cucumber Fruit: Present only 9/9 (3 days pre-Rain 1)** | | | | |
| --- | --- | --- | --- | --- |
| **Phylum** | **Class** | **Order** | **Family** | **Genus** |
| Actinobacteria | Actinobacteria | Actinomycetales | Microbacteriaceae | Microbacterium |
| Proteobacteria | Alphaproteobacteria | Sphingomonadales | Sphingomonadaceae | Sphingomonas |
|  |  |  |  |  |
|  |  |  |  |  |
|  |  |  |  |  |
| **Cucumber Fruit: Present only 9/13 (1 day post-Rain 1)** | | | | |
| **Phylum** | **Class** | **Order** | **Family** | **Genus** |
| Bacteroidetes | Sphingobacteriia | Sphingobacteriales | Sphingobacteriaceae | Sphingobacterium |
| Firmicutes | Bacilli | Bacillales | Paenibacillaceae | Unclassified |
| Proteobacteria | Alphaproteobacteria | Caulobacterales | Caulobacteraceae | Unclassified |
|  |  | Rhizobiales | Aurantimonadaceae | Unclassified |
|  |  | Sphingomonadales | Sphingomonadaceae | Sphingomonas |
|  |  |  |  | Unclassified |
|  | Betaproteobacteria | Burkholderiales | Comamonadaceae | Unclassified |
|  |  |  |  | Unclassified |
|  |  |  | Oxalobacteraceae | Unclassified |
|  |  | Rhodocyclales | Rhodocyclaceae | Unclassified |
|  |  | Unclassified | Unclassified | Unclassified |
|  | Gammaproteobacteria | Enterobacteriales | Enterobacteriaceae | Unclassified |
|  |  |  |  | Unclassified |
|  |  |  |  | Unclassified |
|  |  |  |  | Unclassified |
|  |  |  |  | Unclassified |
|  |  |  |  | Unclassified |
|  |  |  |  | Unclassified |
|  |  |  |  | Unclassified |
|  |  |  |  | Unclassified |
|  |  |  |  | Unclassified |
|  |  |  |  | Unclassified |
|  |  |  |  | Unclassified |
|  |  |  |  | Unclassified |
|  |  | Pseudomonadales | Moraxellaceae | Acinetobacter |
|  |  |  | Pseudomonadaceae | Pseudomonas |
|  |  |  |  |  |
|  |  |  |  |  |
|  |  |  |  |  |
|  |  |  |  | Unclassified |
|  |  | Unclassified | Unclassified | Unclassified |
|  |  | Xanthomonadales | Xanthomonadaceae | Stenotrophomonas |
|  |  |  |  |  |
|  |  |  |  | Unclassified |
|  |  |  |  | Unclassified |
|  |  |  |  | Unclassified |
|  |  |  |  | Unclassified |
|  |  |  |  | Unclassified |
|  |  |  |  | Unclassified |
|  |  |  |  |  |
| **Cucumber Fruit: Present only 9/17 (5 days post-Rain 1)** | | | | |
| **Phylum** | **Class** | **Order** | **Family** | **Genus** |
| Actinobacteria | Actinobacteria | Actinomycetales | Microbacteriaceae | Microbacterium |
|  |  |  |  | Unclassified |
| Proteobacteria | Alphaproteobacteria | Rhizobiales | Methylobacteriaceae | Methylobacterium |
|  |  |  | Rhizobiaceae | Agrobacterium |
|  |  |  |  |  |
|  | Betaproteobacteria | Burkholderiales | Oxalobacteraceae | Unclassified |
|  | Gammaproteobacteria | Pseudomonadales | Pseudomonadaceae | Pseudomonas |
|  |  |  |  | Unclassified |
|  |  |  |  |  |
| **Cucumber Fruit: Present only 9/9 and 9/13 (1 day pre-Rain 1 and 1 day post-Rain 1)** | | | | |
| **Phylum** | **Class** | **Order** | **Family** | **Genus** |
| Proteobacteria | Alphaproteobacteria | Rhizobiales | Methylobacteriaceae | Unclassified |
|  |  |  | Rhizobiaceae | Agrobacterium |
|  |  | Sphingomonadales | Sphingomonadaceae | Novosphingobium |
|  |  |  |  | Sphingomonas |
|  |  |  |  |  |
|  |  |  |  |  |
|  | Gammaproteobacteria | Enterobacteriales | Enterobacteriaceae | Unclassified |
|  |  |  |  |  |
| **Cucumber Fruit: Present only 9/9 and 9/17 (1 day pre-Rain 1 and 5 days post-Rain 1)** | | | | |
| **Phylum** | **Class** | **Order** | **Family** | **Genus** |
| Actinobacteria | Actinobacteria | Actinomycetales | Microbacteriaceae | Microbacterium |
|  |  |  |  |  |
| Proteobacteria | Alphaproteobacteria | Sphingomonadales | Sphingomonadaceae | Sphingomonas |
|  |  |  |  |  |
| **Cucumber Fruit: Present only 9/13 and 9/17 (1 and 5 days post-Rain 1)** | | | | |
| **Phylum** | **Class** | **Order** | **Family** | **Genus** |
| Actinobacteria | Actinobacteria | Actinomycetales | Beutenbergiaceae | Unclassified |
|  |  |  | Microbacteriaceae | Agrococcus |
|  |  |  |  | Leucobacter |
|  |  |  |  | Microbacterium |
|  |  |  |  | Unclassified |
| Proteobacteria | Alphaproteobacteria | Rhizobiales | Rhizobiaceae | Agrobacterium |
|  |  | Rhodobacterales | Rhodobacteraceae | Paracoccus |
|  |  |  |  | Rhodobacter |
|  |  |  |  |  |
|  |  |  |  |  |
|  |  |  |  |  |
|  |  |  |  | Unclassified |
|  |  | Sphingomonadales | Sphingomonadaceae | Sphingomonas |
|  |  |  |  |  |
|  |  |  |  | Unclassified |
|  |  |  |  | Unclassified |
|  | Betaproteobacteria | Burkholderiales | Comamonadaceae | Unclassified |
|  |  |  | Comamonadaceae | Unclassified |
|  |  |  | Oxalobacteraceae | Unclassified |
|  |  |  |  | Unclassified |
|  |  |  |  | Unclassified |
|  |  |  |  | Unclassified |
|  | Gammaproteobacteria | Enterobacteriales | Enterobacteriaceae | Erwinia |
|  |  |  |  | Unclassified |
|  |  |  |  | Unclassified |
|  |  |  |  | Unclassified |
|  |  |  |  | Unclassified |
|  |  |  |  | Unclassified |
|  |  |  |  | Unclassified |
|  |  |  |  | Unclassified |
|  |  | Pseudomonadales | Moraxellaceae | Unclassified |
|  |  |  | Pseudomonadaceae | Pseudomonas |
|  |  |  |  |  |
|  |  |  |  |  |
|  |  | Xanthomonadales | Xanthomonadaceae | Unclassified |
|  |  |  |  |  |
| **Cucumber Fruit: Present in all samples collected across all dates (9/9, 9/13, 9/17)** | | | | |
| **Phylum** | **Class** | **Order** | **Family** | **Genus** |
| Actinobacteria | Actinobacteria | Actinomycetales | Microbacteriaceae | Microbacterium |
|  |  |  |  |  |
|  |  |  |  |  |
| Proteobacteria | Alphaproteobacteria | Rhizobiales | Aurantimonadaceae | Unclassified |
|  |  |  |  | Unclassified |
|  |  |  |  | Unclassified |
|  |  |  | Methylobacteriaceae | Methylobacterium |
|  |  |  |  |  |
|  |  |  |  |  |
|  |  |  | Rhizobiaceae | Agrobacterium |
|  |  |  |  |  |
|  |  |  |  |  |
|  |  |  |  |  |
|  |  |  | Unclassified | Unclassified |
|  |  | Sphingomonadales | Sphingomonadaceae | Sphingomonas |
|  |  |  |  |  |
|  |  |  |  |  |
|  |  |  |  |  |
|  |  |  |  |  |
|  |  |  |  | Unclassified |
|  | Betaproteobacteria | Burkholderiales | Alcaligenaceae | Unclassified |
|  |  |  | Oxalobacteraceae | Unclassified |
|  | Gammaproteobacteria | Enterobacteriales | Enterobacteriaceae | Unclassified |
|  |  |  |  | Unclassified |
|  |  |  |  | Unclassified |
|  |  |  |  | Unclassified |
|  |  | Pseudomonadales | Moraxellaceae | Unclassified |
|  |  |  |  | Unclassified |
|  |  |  | Pseudomonadaceae | Pseudomonas |
|  |  |  |  |  |
|  |  |  |  |  |
|  |  |  |  |  |
|  |  |  |  |  |
|  |  |  |  |  |
|  |  |  |  | Unclassified |
|  |  |  |  | Unclassified |
|  |  |  | Unclassified | Unclassified |
|  |  | Xanthomonadales | Xanthomonadaceae | Unclassified |
|  |  |  |  |  |
|  |  |  |  |  |
| **Tomato Fruit: Present only 9/9 (3 days pre-Rain 1)** | | | | |
| **Phylum** | **Class** | **Order** | **Family** | **Genus** |
| Actinobacteria | Actinobacteria | Actinomycetales | Microbacteriaceae | Agrococcus |
| Proteobacteria | Alphaproteobacteria | Rhizobiales | Methylobacteriaceae | Unclassified |
|  | Gammaproteobacteria | Pseudomonadales | Pseudomonadaceae | Pseudomonas |
|  |  |  |  |  |
| **Tomato Fruit: Present only 9/13 (1 day post-Rain 1)** | | | | |
| **Phylum** | **Class** | **Order** | **Family** | **Genus** |
| Actinobacteria | Actinobacteria | Actinomycetales | Microbacteriaceae | Microbacterium |
| Proteobacteria | Alphaproteobacteria | Caulobacterales | Caulobacteraceae | Unclassified |
|  |  | Rhizobiales | Rhizobiaceae | Agrobacterium |
|  |  |  |  |  |
|  |  | Rhodobacterales | Rhodobacteraceae | Rhodobacter |
|  |  |  |  |  |
|  |  |  |  | Unclassified |
|  |  | Sphingomonadales | Sphingomonadaceae | Sphingomonas |
|  | Betaproteobacteria | Burkholderiales | Oxalobacteraceae | Unclassified |
|  |  |  |  | Unclassified |
|  |  | Unclassified | Unclassified | Unclassified |
|  | Gammaproteobacteria | Enterobacteriales | Enterobacteriaceae | Unclassified |
|  |  |  |  | Unclassified |
|  |  |  |  | Unclassified |
|  |  |  |  | Unclassified |
|  |  |  |  | Unclassified |
|  |  | Pseudomonadales | Pseudomonadaceae | Pseudomonas |
|  |  |  |  |  |
|  |  |  |  |  |
|  |  |  | Unclassified | Unclassified |
|  |  | Xanthomonadales | Xanthomonadaceae | Stenotrophomonas |
|  |  |  |  |  |
|  |  |  |  | Unclassified |
|  |  |  |  | Unclassified |
|  |  |  |  |  |
| **Tomato Fruit: Present only 9/17 (5 days post-Rain 1)** | | | | |
| **Phylum** | **Class** | **Order** | **Family** | **Genus** |
| Actinobacteria | Actinobacteria | Actinomycetales | Microbacteriaceae | Unclassified |
|  |  |  | Unclassified | Unclassified |
| Proteobacteria | Alphaproteobacteria | Rhizobiales | Methylobacteriaceae | Unclassified |
|  |  |  | Rhizobiaceae | Agrobacterium |
|  |  |  |  | Unclassified |
|  |  | Rhodobacterales | Rhodobacteraceae | Paracoccus |
|  | Betaproteobacteria | Burkholderiales | Comamonadaceae | Unclassified |
|  | Gammaproteobacteria | Pseudomonadales | Pseudomonadaceae | Pseudomonas |
|  |  | Xanthomonadales | Xanthomonadaceae | Stenotrophomonas |
|  |  |  |  |  |
| **Tomato Fruit: Present only 9/9 and 9/13 (1 day pre-Rain 1 and 1 day post-Rain 1)** | | | | |
| **Phylum** | **Class** | **Order** | **Family** | **Genus** |
| Actinobacteria | Actinobacteria | Actinomycetales | Microbacteriaceae | Microbacterium |
|  |  |  |  |  |
| **Tomato Fruit: Present only 9/9 and 9/17 (1 day pre-Rain 1 and 5 days post-Rain 1)** | | | | |
| **Phylum** | **Class** | **Order** | **Family** | **Genus** |
| Actinobacteria | Actinobacteria | Actinomycetales | Microbacteriaceae | Microbacterium |
| Proteobacteria | Alphaproteobacteria | Rhizobiales | Rhizobiaceae | Agrobacterium |
|  |  |  |  |  |
|  |  | Sphingomonadales | Sphingomonadaceae | Sphingomonas |
|  | Gammaproteobacteria | Pseudomonadales | Pseudomonadaceae | Pseudomonas |
|  |  |  |  |  |
|  |  |  |  |  |
| **Tomato Fruit: Present only 9/13 and 9/17 (1 and 5 days post-Rain 1)** | | | | |
| **Phylum** | **Class** | **Order** | **Family** | **Genus** |
| Actinobacteria | Actinobacteria | Actinomycetales | Microbacteriaceae | Agrococcus |
|  |  |  |  | Leucobacter |
|  |  |  |  | Microbacterium |
|  |  |  |  | Mycetocola |
| Proteobacteria | Alphaproteobacteria | Rhizobiales | Methylobacteriaceae | Methylobacterium |
|  |  |  |  |  |
|  |  |  |  | Unclassified |
|  |  |  | Rhizobiaceae | Agrobacterium |
|  |  |  |  |  |
|  |  |  |  |  |
|  | Betaproteobacteria | Burkholderiales | Comamonadaceae | Unclassified |
|  |  |  | Oxalobacteraceae | Unclassified |
|  | Gammaproteobacteria | Enterobacteriales | Enterobacteriaceae | Erwinia |
|  |  |  |  | Unclassified |
|  |  |  |  | Unclassified |
|  |  | Pseudomonadales | Pseudomonadaceae | Pseudomonas |
|  |  |  |  | Unclassified |
|  |  | Xanthomonadales | Xanthomonadaceae | Unclassified |
|  |  |  |  |  |
| **Tomato Fruit: Present in all samples collected across all dates surrounding Rain 1 (9/9, 9/13, 9/17)** | | | | |
| **Phylum** | **Class** | **Order** | **Family** | **Genus** |
| Actinobacteria | Actinobacteria | Actinomycetales | Beutenbergiaceae | Unclassified |
|  |  |  | Microbacteriaceae | Curtobacterium |
|  |  |  |  | Microbacterium |
|  |  |  |  |  |
|  |  |  |  |  |
|  |  |  |  | Unclassified |
| Proteobacteria | Alphaproteobacteria | Rhizobiales | Methylobacteriaceae | Methylobacterium |
|  |  |  |  | Unclassified |
|  |  |  |  | Unclassified |
|  |  |  | Rhizobiaceae | Agrobacterium |
|  |  |  |  |  |
|  |  |  |  |  |
|  |  |  |  |  |
|  |  |  | Unclassified | Unclassified |
|  |  | Sphingomonadales | Sphingomonadaceae | Sphingomonas |
|  |  |  |  |  |
|  |  |  |  |  |
|  |  |  |  | Unclassified |
|  | Betaproteobacteria | Burkholderiales | Alcaligenaceae | Unclassified |
|  |  |  | Comamonadaceae | Unclassified |
|  | Gammaproteobacteria | Enterobacteriales | Enterobacteriaceae | Unclassified |
|  |  |  |  | Unclassified |
|  |  |  |  | Unclassified |
|  |  | Pseudomonadales | Moraxellaceae | Unclassified |
|  |  |  | Pseudomonadaceae | Pseudomonas |
|  |  |  |  |  |
|  |  |  |  |  |
|  |  |  |  |  |
|  |  |  |  |  |
|  |  |  |  |  |
|  |  |  |  |  |
|  |  |  |  | Unclassified |
|  |  |  |  | Unclassified |
|  |  |  |  | Unclassified |
|  |  |  | Unclassified | Unclassified |
|  |  | Xanthomonadales | Xanthomonadaceae | Unclassified |
|  |  |  |  | Unclassified |
|  |  |  |  | Unclassified |
|  |  |  |  | Unclassified |
